# The influenza virus hemagglutinin head evolves faster than the stalk domain

**DOI:** 10.1038/s41598-018-28706-1

**Published:** 2018-07-11

**Authors:** Ericka Kirkpatrick, Xueting Qiu, Patrick C. Wilson, Justin Bahl, Florian Krammer

**Affiliations:** 1 Department of Microbiology, Icahn School of Medicine at Mount Sinai, New York, NY USA; 2 Graduate School of Biomedical Sciences, Icahn School of Medicine at Mount Sinai, New York, NY USA; 3 University of Texas School of Public Health, Houston, TX USA; 4 Department of Medicine, Section of Rheumatology, Gwen Knapp Center for Lupus and Immunology Research, University of Chicago, Chicago, IL USA; 5 Program in Emerging Infectious Diseases, Duke-National University of Singapore Graduate Medical School, Singapore, Singapore

## Abstract

The limited ability of current influenza virus vaccines to protect from antigenically drifted or shifted viruses creates a public health problem that has led to the need to develop effective, broadly protective vaccines. While current influenza virus vaccines mostly induce an immune response against the immunodominant and variable head domain of the hemagglutinin, the major surface glycoprotein of the virus, the hemagglutinin stalk domain has been identified to harbor neutralizing B-cell epitopes that are conserved among and even between influenza A virus subtypes. A complete understanding of the differences in evolution between the main target of current vaccines and this more conserved stalk region are missing. Here, we performed an evolutionary analysis of the stalk domains of the hemagglutinin of pre-pandemic seasonal H1N1, pandemic H1N1, seasonal H3N2, and influenza B viruses and show quantitatively for the first time that the stalk domain is evolving at a rate that is significantly slower than that of the head domain. Additionally, we found that the cross-reactive epitopes in the stalk domain targeted by broadly neutralizing monoclonal antibodies are evolving at an even slower rate compared to the full head and stalk regions of the protein. Finally, a fixed-effects likelihood selection analysis was performed for these virus groups in both the head and stalk domains. While several positive selection sites were found in the head domain, only a single site in the stalk domain of pre-pandemic seasonal H1 hemagglutinin was identified at amino acid position 468 (H1 numbering from methionine). This site is not located in or close to the epitopes of cross-reactive anti-stalk monoclonal antibodies. Furthermore, we found that changes in this site do not significantly impact virus binding or neutralization by human anti-stalk antibodies, suggesting that some positive selection in the stalk domain is independent of immune pressures. We conclude that, while the stalk domain does evolve over time, this evolution is slow and, historically, is not directed to aid in evading neutralizing antibody responses.

## Introduction

**Figure 1:**
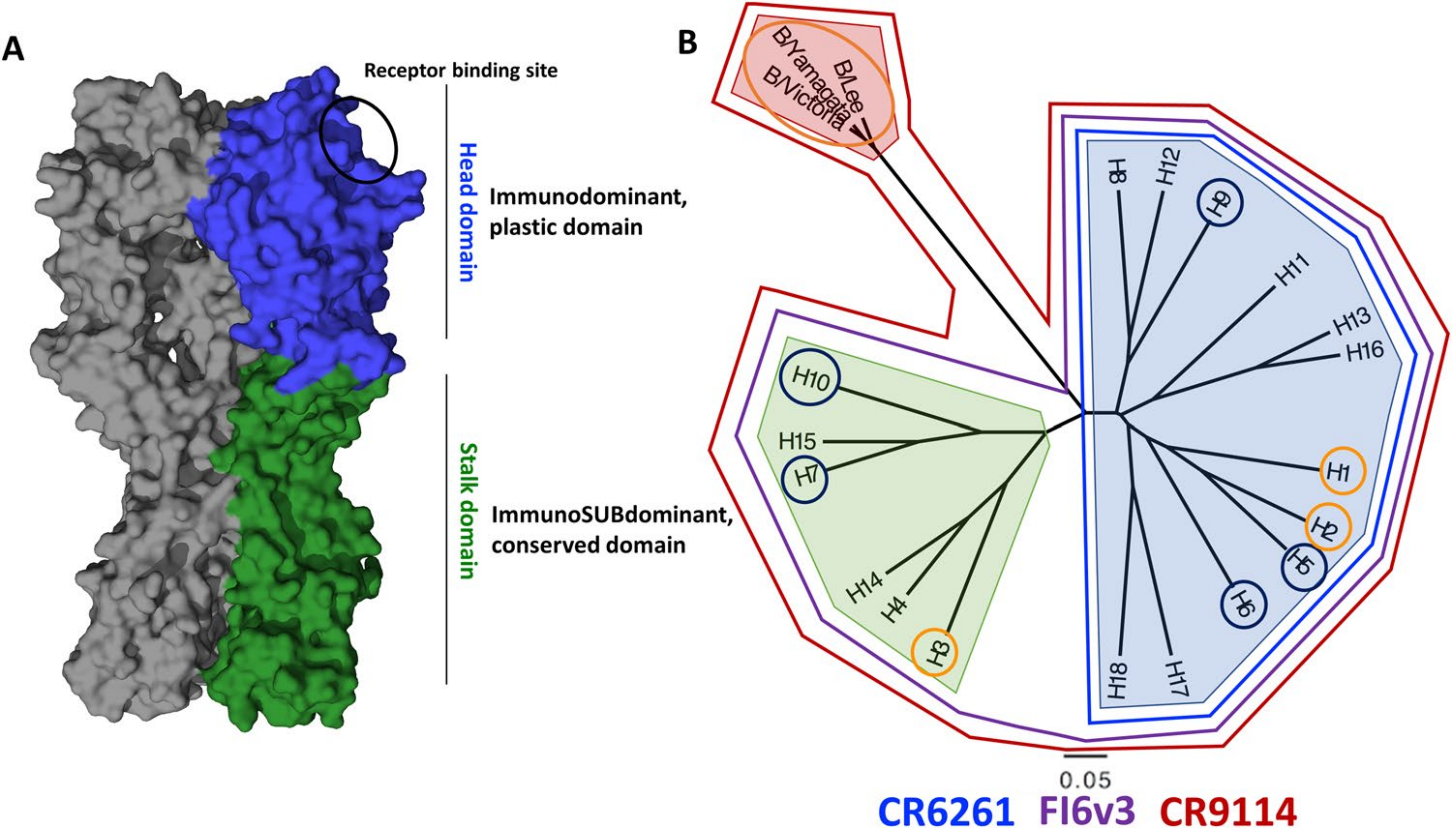
Structure and classification of influenza virus hemagglutinins (HA). (**A**) The homotrimeric structure of the A/Puerto Rico/8/1934 hemagglutinin (PDB ID 1RU7,^74^). A monomer of the stalk domain is colored in green, while a monomer of the head domain is colored in blue. The receptor binding site is circled in black. (**B**) Phylogenetic tree of all known hemagglutinin subtypes of influenza A viruses and influenza B virus HA lineages. The light blue shading shows influenza A virus group 1 HAs, light green influenza A virus group 2 HAs, and light red shows influenza B virus HAs. HAs circled in orange are currently circulating in humans (or have in the past like H2) while those in dark blue have infected humans, but mostly reside in avian hosts. The binding breadth of broadly neutralizing anti-stalk mAbs CR6261, CR9114 and FI6v3 is outlined.

Influenza virus infections are a major public health concern, affecting between 10 and 20% of the human population annually and causing significant morbidity and mortality worldwide^1^. The influenza virus is an RNA virus that undergoes constant antigenic drift, therefore current vaccines have to be re-formulated and re-administered on an annual basis to maintain efficacy. Unfortunately, the selected vaccine strains do not always match the circulating pathogenic strains and this problem causes low and unpredictable vaccine effectiveness that ranges from approximately 10% to 60%^2^. Furthermore, seasonal vaccination does not protect against newly emerging pandemic and zoonotic influenza viruses. Universal/broadly protective influenza virus vaccines that are unaffected by antigenic drift would alleviate the burden of seasonal influenza virus infections as well as annual re-formulations and re-administrations of vaccines and would significantly enhance pandemic preparedness. Several of these novel vaccine approaches focus on targeting a more conserved region of the influenza virus, the stalk domain of the hemagglutinin (HA) glycoprotein of the virion^3^ (Fig. 1A).

Influenza viruses are members of the Orthomyxoviridae family and are phylogenetically grouped into four virus genera, influenza A, B, C and D^4,5^. Influenza A viruses are additionally grouped based on the sequence and antigenic relatedness of their HA into influenza A virus group 1 (H1, H2, H5, H6, H8, H9, H11, H12, H13, H16, HL17 and HL18) and influenza A virus group 2 (H3, H4, H7, H10, H14, and H15). Influenza B viruses have diverged from the ancestral B/Lee/1940 strain into two distinct co-circulating lineages, referred to as B/Yamagata/16/88-like and B/Victoria/2/87-like viruses^3,4^ (Fig. 1B). H1N1, H3N2 and the influenza B lineages are the virus types that are currently circulating in humans, causing seasonal outbreaks. Additionally, H2N2 has previously circulated in humans, but no longer causes seasonal outbreaks since it was replaced in 1968 by H3N2. H5, H6, H7, H9 and H10 viruses circulate mostly in avian species, but have the potential to cause zoonotic infections in humans^6–10^ (Fig. 1B). The immunodominant HA globular head domain has high plasticity, with distinct antigenic sites undergoing constant antigenic drift. The immunosubdominant stalk domain is relatively conserved among subtypes, making it a better target for broadly protective antibodies^4,11^. This is illustrated by the binding footprint of broadly neutralizing anti-stalk monoclonal antibodies (mAbs) such as CR6261 (pan-group 1 mAb), FI6v3 (pan-influenza A mAb), and CR9114 (pan-influenza A and B mAb)^12–14^, whose breadth of binding is outlined in Fig. 1B.

Several vaccination strategies have been developed to attempt to refocus the immune response from the immunodominant head domain towards the immunosubdominant stalk domain. These include chimeric HA based approaches as well as headless HA based immunogens^15–17^. Antibodies generated against the stalk have the potential to be cross-reactive between influenza virus strains within and across subtypes and to protect against a broad range of influenza viruses. In addition to universal and broadly protective influenza virus vaccines, several anti-stalk monoclonal antibodies are currently in clinical development as influenza virus therapeutics^18,19^. If the right epitopes are targeted, these vaccines and therapeutics could be broadly protective for extended periods of time^15–17,20,21^. Therefore, quantifying the evolutionary rates of the head and stalk domains of the hemagglutinin protein individually might inform about the success of future stalk-based vaccine approaches. Here we analyze both evolutionary rates and positive selection of the head and stalk domains of HAs from pre-pandemic seasonal H1N1 (sH1N1), pandemic H1N1 (pH1N1), H3N2 and the two co-circulating influenza B lineages.

## Results

### A partitioning scheme for determining head and stalk domain evolutionary rates

We chose to calculate the evolutionary rates of the five types of influenza viruses that are circulating or circulated until recently in humans: pre-pandemic seasonal H1N1 (sH1N1, 1918–1957 and 1977–2009), pandemic H1N1 (pH1N1, 2009–2017), H3N2 (1968–2017), B/Victoria/2/87-like lineage (B/Vic, 1987–2017), and B/Yamagata/16/88-like lineage (B/Yam, 1988–2017). For subsequent analyses, we included isolate sequences from all years in which a particular subtype circulated, despite variation in passaging history for older isolates. Even though passaged viruses may introduce a few false positive selection signals (from cell or egg adaptations), removing these sequences would increase each analyses’ phylogenetic uncertainty and lead us to exclude important information in the shared ancestry of the viral population, which is especially important given the large number of parameters in our partitioning scheme^22,23^. To verify that this reasoning was appropriate, we compared the nonsynonymous rates (dN) between sH1N1 sequences from isolates that were not passaged to isolates with mixed passaging histories and found a high correlation (0.76 correlation coefficient) between the two datasets suggesting minimal effects of including earlier, passaged isolates in our analyses (see methods and Fig. S3).

**Figure 2:**
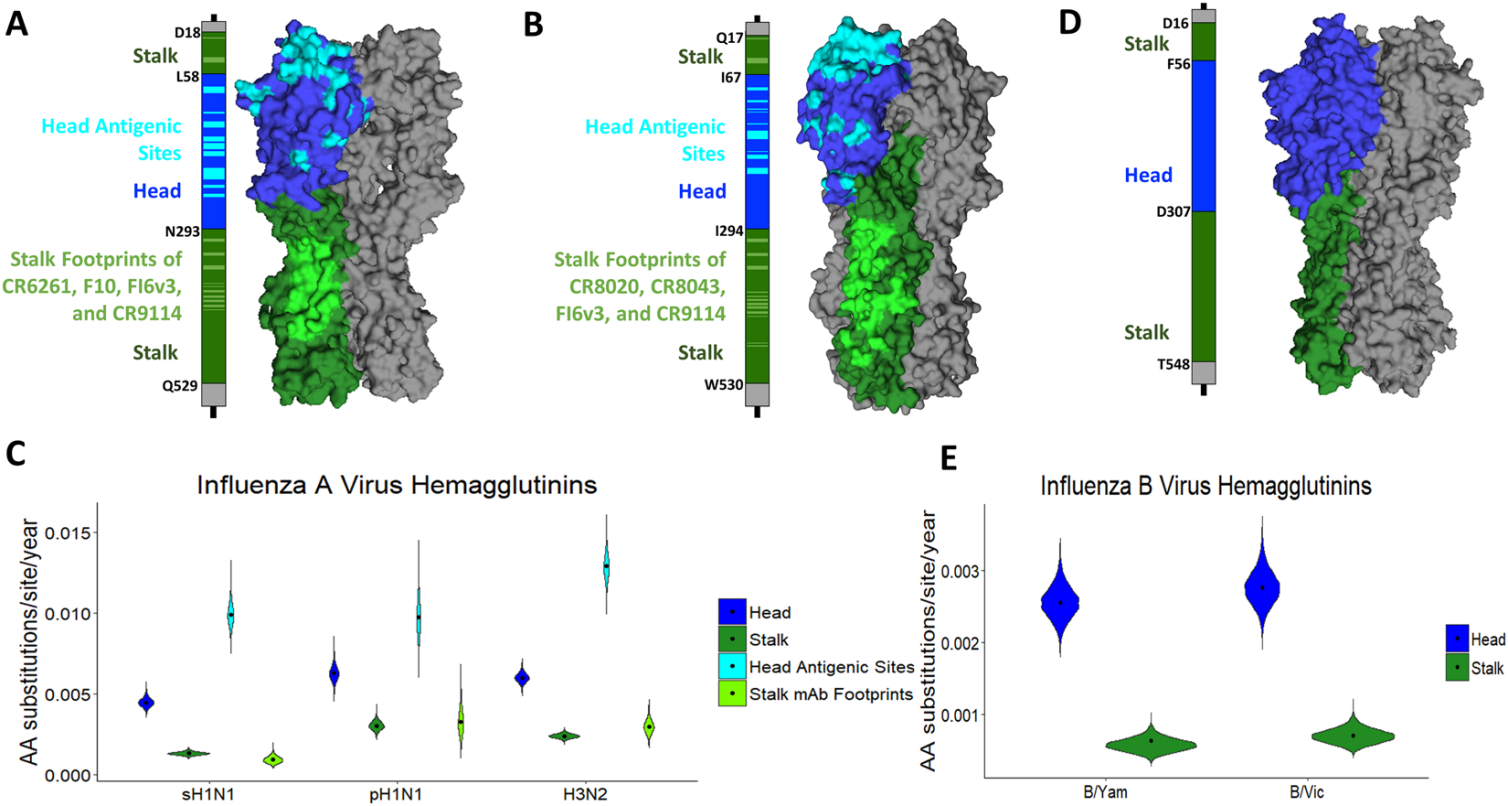
Determining the evolutionary rates of head antigenic sites and stalk monoclonal antibody footprints for influenza A viruses. (**A**) Linear schematic of the H1 HA molecule and partitions used for analysis. The stalk domain amino acid demarcations are shown on the figure (D18 to L58 and N293 to Q529) in green. The head domain (amino acids 59–292) is shown in blue and the signal peptide, cytoplasmic, and transmembrane domains (STC) are shown in grey (amino acids 1–17 and 530–566). Approximate estimation of the stalk mAb footprints are shown in light green while approximate estimates of head antigenic sites are shown in cyan. On the right is the 3D representation of an H1 HA (1RU7,^74^) with the head in blue, head antigenic sites in cyan, stalk in green, and stalk mAb epitopes in light green. (**B**) Linear schematic of the H3 HA molecule and partitions used for analysis. The stalk domain amino acid demarcations are shown on the figure (Q17 to I67 and I294 to W530) in green. The head domain (amino acids 68–293) is shown in blue and the signal peptide, cytoplasmic, and transmembrane domains (STC) are shown in grey (amino acids 1–17 and 531–565). Approximate estimation of the stalk mAb footprints are shown in light green while approximate estimates of head antigenic sites are shown in cyan. On the right is the 3D representation of a H3 HA (2YPG,^75^) with the head in blue, head antigenic sites in cyan, stalk in green, and stalk mAb epitopes in light green. (**C**) Evolutionary rates of sH1N1, pH1N1, and H3N2 virus hemagglutinin head (blue), stalk (dark green), head antigenic sites (cyan) and stalk mAb footprints (green). The mean and 95% credible intervals of BEAST runs (using a single dataset) are shown in amino acid substitutions/site/year (a/s/t). (**D**) Linear schematic of the influenza B virus HA molecule and partitions used for analysis. The stalk domain amino acid demarcations are shown on the figure (D16 to F56 and D307 to T548) in green. The head domain (amino acids 57–306) is shown in blue and the signal, cytoplasmic, and transmembrane domains (STC) are shown in grey (amino acids 1–15 and 549–583). On the right is the 3D representation of an influenza B virus HA (4M40,^76^) with the head in blue and the stalk in green. (**E**) Evolutionary rates of B/Victoria/2/87-like and B/Yamagata/16/88-like virus hemagglutinin head (blue) and stalk (dark green). The mean and 95% credible intervals of BEAST runs (using a single dataset) are shown in amino acid substitutions/site/year (a/s/t).

To estimate head and stalk domain evolutionary rates we used a structurally informed partitioning scheme that allows calculating rate variations between the two domains^23^. We used an approximate codon model (SRD06) to account for the degenerate property of the third codon position. The signal peptide, transmembrane region, and cytoplasmic domain (STC) of the HA protein were grouped into their own non-codon-specific parameter due to the limitations in reliable parameter estimates from a short nucleotide region^23^. For the purpose of this study, the head domain was defined as ranging from cysteine 52 (C52) to cysteine 277 (C277) in the traditional H3 numbering. However, for our analyses the head and stalk domains were determined based on a multiple sequence alignment of the aforementioned head domain to A/South Carolina/1/1918 for H1N1, A/Aichi/2/1968 for H3N2, and B/Yamagata/16/1988 for influenza B viruses and their previously defined regions, with numbering beginning from methionine, not traditional H3 numbering^24^. Numbering for sH1N1 strains containing a deletion outside the receptor binding site (at residue K147 in A/South Carolina/1/1918) was kept consistent with other strains by using a multiple sequence alignment to determine head and stalk regions. Analysis included the entire head from C59 to C292 for H1N1 viruses, C68 to C293 for H3N2 viruses, and the respective A57 and A306 for influenza B viruses (Fig. 2A,B and D). The stalk domain includes two portions, from D18 to L58 (the N-terminus of HA1) and N293 to Q529 (the C-terminus of HA1 plus the ectodomain of HA2) for H1N1 viruses, Q17 to I67 and I294 to W530 for H3N2 viruses, and D16 to F56 and D307 to T548 for influenza B viruses. We conducted likelihood ratio testing to determine if using a structurally informed partitioning scheme increased evolutionary rate accuracy by generating a test statistic equal to: 2 × [ln(likelihood for structurally informed partitioning) – ln(likelihood for no partitioning)]. The significance of the test statistic was determined with a Chi-square table using 2 degrees of freedom. These results, included in Table S1, indicate that adding STC, head, and stalk domain partitions significantly increase the likelihood of each analysis. In line with previous studies, our results indicate that the nucleotide substitution rates for the head domain were faster than for the stalk domain^23^ (Fig. S1, Table S2). To additionally validate these nucleotide results, we used Bayes Factors (BF) as statistical analyses to determine if the different nucleotide rates were significantly different. Bayes Factors were calculated by comparing the posterior odds of the Pr(Head > Stalk)/(Pr(Stalk > Head) divided by the prior odds for each state of the Markov chain Monte Carlo (MCMC) generated through our analysis of nucleotide data. Typically, BF are interpreted to be nonsignificant if less than 1 and marginally significant if greater than 3, with more significance being attributed to higher values^25^. Our results demonstrate that for all influenza types analyzed there was very strong to decisive significance that the head evolves faster than the stalk domain (Table S2).

### Determining evolutionary rates of head antigenic sites and stalk mAb footprints

While changes at the nucleotide level are phylogenetically informative, changes at the amino acid level are important for evasion of antibody-recognition. First, we modeled amino acid substitutions of the head domain and stalk domain by using the set of trees obtained through our analysis of nucleotide data. Unsurprisingly, the head domain had a higher amino acid substitution rate than the stalk (Fig. 2C,D and Table 1).

**Table 1:** Summary of evolutionary rates and selection analysis of head and stalk domains of sH1N1, pH1N1, H3N2, B/Yam and B/Vic based on empirical trees.

Virus	Head Evolutionary Rate (95% BCI*) (a/s/y)	Stalk Evolutionary Rate (95% BCI*) (a/s/y)	Head:Stalk Rate Ratio	Overall ω	Head ω	Stalk ω	Head:Stalk ω Ratio	Head:Stalk Approximate ω Ratio^δ^ (95% BCI*)
sH1N1	4.46 × 10^−3^ (4.38 × 10^−3^– 4.54 × 10^−3^)	1.30 × 10^−3^ (1.24 × 10^−3^– 1.35 × 10^−3^)	3.4	0.32	0.44	0.13	3.2	3.0 (2.2–3.8)
pH1N1	6.26 × 10^−3^ (6.15 × 10^−3^– 6.37 × 10^−3^)	3.02 × 10^−3^ (2.92 × 10^−3^– 3.12 × 10^−3^)	2.1	0.22	0.28	0.16	1.7	1.6 (1.2–2.2)
H3N2	5.99 × 10^−3^ (5.92 × 10^−3^– 6.06 × 10^−3^)	2.38 × 10^−3^ (2.32 × 10^−3^– 2.44 × 10^−3^)	2.5	0.31	0.48	0.16	3.0	2.2 (1.7–2.7)
B/Yam	2.55 × 10^−3^ (2.46 × 10^−3^– 2.64 × 10^−3^)	5.84 × 10^−4^ (5.07 × 10^−4^– 6.61 × 10^−4^)	4.4	0.15	0.21	0.05	4.3	3.4 (2.1–4.9)
B/Vic	2.76 × 10^−3^ (2.66 × 10^−3^– 2.85 × 10^−3^)	7.06 × 10^−4^ (6.30 × 10^−4^– 7.81 × 10^−4^)	3.9	0.13	0.26	0.06	4.2	3.3 (2.2–4.7)

The approximate dN/dS rate ratio is calculated using the relative substitution rates for codon positions 1 + 2/codon position 3 for the head and stalk domains for each state of the Markov chain Monte Carlo (MCMC).

^*^Bayesian Credible Interval. ^**δ**^The Approximate **ω** is a ratio of the substitution rate for codon positions 1 + 2 divided by the substitution rate for codon position 3, for both the head and stalk domains.

**Figure 3:**
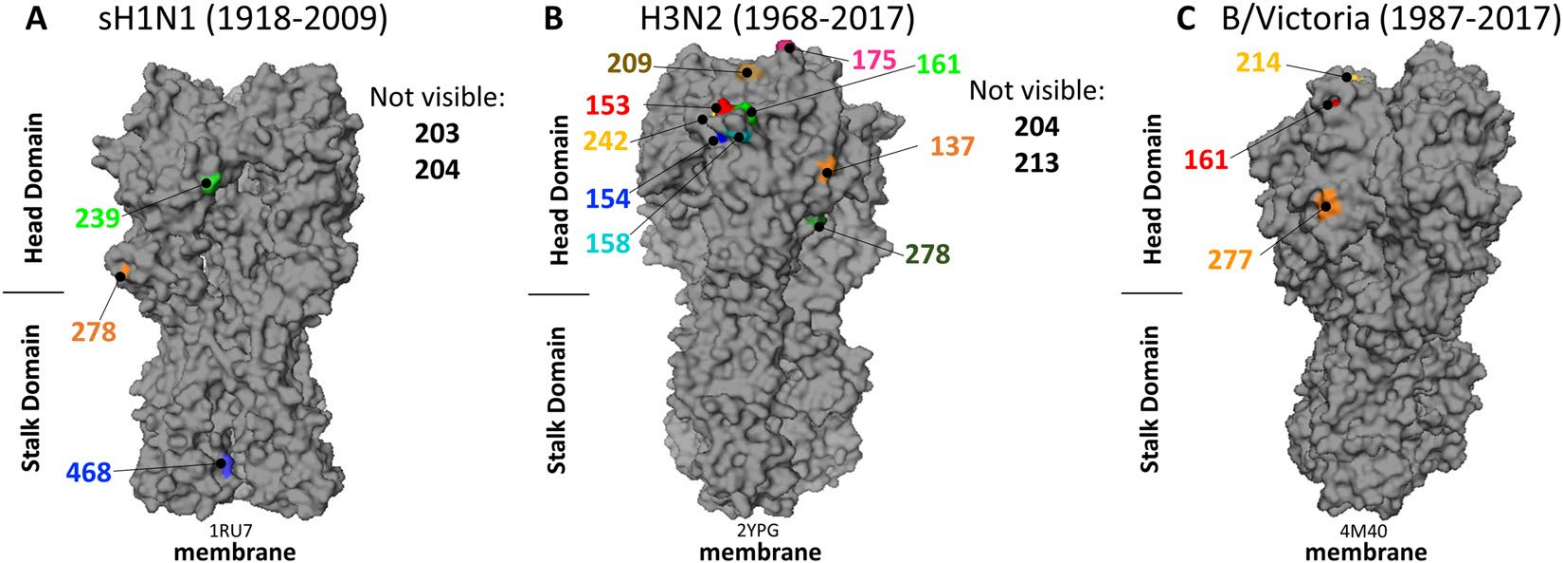
Data Monkey fixed-effects likelihood selection analysis results. (**A**) sH1N1 HA protein (PDB ID 1RU7,^74^) showing 4 positive selection sites in the head domain and 1 positive selection site in the stalk domain. **(B)** H3N2 HA (PDB ID 2YPG,^75^) showing 11 positive selection sites in the head domain and no selection sites in the stalk domain. **(C)** B/Victoria HA (PDB ID 4M40,^76^) showing the 2 positive selection sites for B/Victoria in the head domain and 1 positive selection site for B/Yamagata (in yellow) and no selection sites in the stalk domain. Some sites are on the interior of the HA and are listed as “not visible”.

Additionally, we investigated how the antigenic sites in the head domain and the footprints of stalk binding cross-reactive antibodies vary in their estimated mean amino acid substitution rates to see if there are significant differences between sites that are assumed to be the primary target of a seasonal vaccination strategy (antigenic sites of the H1 and H3 head domain) and sites that would be targeted by a stalk-based vaccination strategy (stalk mAb footprints). For this analysis, the H1 head antigenic sites were chosen based on their classical definitions as described in detail by Caton *et al*., Garcia-Barreno *et al*., Matsuzaki *et al*., and Manicassamy *et al*. and are illustrated in Fig. S2A^26–29^. The ‘classical’ antigenic sites were historically determined using murine mAbs and analysis of changes in amino acid sequences connected to antigenic drift (as measured by reduction of HI activity). The H3 head antigenic sites were chosen based on their classical definitions by Underwood and Wiley *et al*. and are illustrated in Fig. S3B^30,31^. These ‘classical’ antigenic sites were identified based on their connection with antigenic drift and by using strain specific murine mAbs on several early circulating strains of H3N2. Since the HA stalk is not directly involved in classical antigenic drift or HI activity^32^, a different approach was chosen to identify antigenic regions of this domain. We considered the footprints of several stalk-targeting cross-reactive mAbs for both H1 HA (CR9114, FI6v3, F10, and CR6261) and H3 HA (CR8020, CR8043, FI6v3, MEDI8852 and CR9114) as sites that would be targeted by an immune response directed towards the stalk domain (Figs 2B,C and 3A–B)^12–14,20,33,34^. The amino acid substitution rates of the head antigenic sites and the stalk mAb footprints indicate that the majority of mutations in the head were focused on sites related to immune escape, while the majority of mutations in the stalk seem to be evenly dispersed throughout the domain (Tables 1, 2). This interpretation comes from the observation that head antigenic sites have much higher substitution rates than the head domain overall, while stalk mAb footprints have similar rates to the entire stalk domain (Fig. 2C,E).

**Table 2:** Summary of evolutionary rates of head antigenic sites and stalk-mAb footprints of sH1N1, pH1N1 and H3N2 based on empirical trees.

Virus	Head Antigenic Sites Evolutionary Rate (a/s/y) (95% BCI*)	Stalk mAb Footprint Evolutionary Rate (a/s/y)	Head Antigenic Sites:Stalk mAb Footprint Rate Ratio
sH1N1	9.90 × 10^−3^ (9.77 × 10^−3^–1.00 × 10^−2^)	9.11 × 10^−4^ (7.83 × 10^−4^–1.04 × 10^−3^)	10.9
pH1N1	9.74 × 10^−3^ (9.54 × 10^−3^–9.97 × 10^−3^)	3.26 × 10^−3^ (3.02 × 10^−3^–3.54 × 10^−3^)	3.0
H3N2	1.29 × 10^−2^ (1.27 × 10^−2^–1.30 × 10^−2^)	2.96 × 10^−3^ (2.82 × 10^−3^–3.10 × 10^−3^)	4.3

^*^Bayesian Credible Interval.

The whole head domain of sH1N1 evolves 3.4 times faster than the stalk domain (4.46 × 10^−3^ a/s/y and 1.30 × 10^−3^ a/s/y, respectively) (Table 1). For sH1N1, head antigenic sites showed an average substitution rate of 9.90 × 10^−3^ amino acid substitutions/site/year (a/s/y) while stalk epitopes had an average rate of 9.11 × 10^−4^ a/s/y, almost 10-fold slower (Fig. 2C, Table 2).

For pH1N1, the head antigenic sites have a slower evolutionary rate than that of sH1N1 (9.74 × 10^−3^ a/s/y) but this rate is still 3.0 times higher than that for the stalk epitopes (3.26 × 10^−3^ a/s/y) (Fig. 2C, Table 2). Additionally, the head antigenic sites are evolving faster than the head domain (6.26 × 10^−3^ a/s/y), while the stalk mAb footprints are evolving at almost the same rate as the whole stalk domain (3.26 × 10^−3^ a/s/y vs 3.02 × 10^−3^ a/s/y) (Tables 1, 2). Interestingly, the evolutionary rates of the head antigenic sites for both sH1N1 and pH1N1 are similar, while the stalk domain rates are very different. As pH1N1 becomes more adapted to its human hosts, by stabilizing HA trimers and by optimally adapting its fusion machinery (both influenced by the stalk domain), it will likely need to accumulate fewer mutations to adapt to growth in human cells and its evolution will likely be more shaped by the human immune response. Therefore, we expect the rates of evolution in the stalk domain to decrease and begin to follow a pattern similar to that of sH1N1.

For H3N2, the head domain is evolving at a rate of 5.99 × 10^−3^ a/s/y while the stalk domain is evolving at a rate of 2.38 × 10^−3^ a/s/y (Fig. 2C, Table 1). Like for H1N1 viruses, the head antigenic sites are evolving at the highest rate (1.29 × 10^−2^ a/s/y) while stalk mAb footprints are evolving at a rate similar to the overall stalk domain (2.96 × 10^−3^ a/s/y) (Table 2, Fig. 2C). As seen in previous studies of the evolutionary diversity of influenza viruses, H3N2 rates are higher than those of sH1N1^35,36^. When comparing H3N2 and pH1N1, the head antigenic sites of H3N2 are evolving slightly faster while the stalk mAb footprints are evolving at almost the same rate.

For influenza B viruses, the head domain is evolving at a similar rate for both lineages (2.76 × 10^−3^ and 2.55 × 10^−3^ for B/Victoria/2/87-like lineage and B/Yamagata/16/88-like lineage viruses, respectively) (Table 1 and Fig. 2E). The stalk domains are also evolving at a similar rate for both lineages but much slower than the head domains (7.06 × 10^−4^ and 5.84 × 10^−4^ for B/Victoria/2/87-like lineage and B/Yamagata/16/88-like lineage viruses, respectively). When comparing these rates to those of influenza A viruses, we see that both domains are evolving more slowly, with the stalk domains having the slowest rates. Additionally, the head to stalk ratios for influenza B viruses are higher than those for both pH1N1 and H3N2 viruses (Table 1) indicating that there is a higher difference in rates of evolution between the head and stalk in influenza B vs influenza A viruses. Overall, we have shown validated amino acid substitution rates for the individual domains of the HA protein and sites targeted by an immunodominant (head antigenic sites) and immunosubdominant (stalk mAb footprints) antibody response.

### Selection analysis shows little positive selection in the stalk domain

Further, we wanted to additionally relate patterns of positive selection to the differences in amino acid substitution rates. To formally assess positive selection on the HA gene, we calculated the overall dN/dS (ω) rate ratio which shows that each virus type tested was under strong purifying selection (Table 1). To determine the relative strength of selection on the respective regions we took two independent approaches. First, we calculated ω for the head domain and the stalk domain for each codon individually from a provided alignment and maximum likelihood tree. The same codons selected for the head and stalk domains in our evolutionary analyses were used determining ω. In order to account for phylogenetic uncertainty, we additionally calculated an approximate dN/dS value as a rate ratio of the nucleotide substitution rate for codon positions 1 + 2 divided by the nucleotide substitution rate for codon position 3, for both the head and stalk domains (Table 1). Overall, sH1N1 had the highest dN/dS ratio of 0.32 while B/Victoria/2/87-like lineage viruses had the lowest dN/dS ratio of 0.13. This result is in line with our findings of lower evolutionary rates for influenza B viruses compared to influenza A viruses. Our results show that the relative selection on the head is substantially greater than the stalk for all influenza virus types tested, despite the HA gene being subjected to very strong purifying selection. The Bayesian credible interval estimated for each influenza virus type analyzed is greater than 1 indicating that despite uncertainty in phylogenetic estimation, the selection pressure on the head is decisively greater than the stalk, which could explain the stark differences in their amino acid substitution rates.

Due to technical limitations of the number of sequences that could be analyzed, we sub-sampled our original data sets three times to have 3 independent tests to ensure that the original data set was adequately represented. The smaller subsamples were also more easily handled by the web server Data Monkey, which was used to conduct the selection analysis in addition to calculating overall ω^37,38^. Only sites that appeared in all three of our subsamples were considered as true positive selection sites. We used a fixed-effects likelihood (FEL) selection analysis to identify positively selected sites. FEL analysis is a more stringent selection analysis than traditional counting methods and determines positive selection by comparing the maximum likelihood of nonsynonymous mutations occurring at a codon to the maximum likelihood of no changes occurring at the codon. If this ratio is determined to be significantly greater than one, it is classified as positive selection. The FEL positive selection method has been shown to have fewer false positive results than other methods, which is why it was chosen for this analysis^39^. We expected to identify positive selection near/in antigenic sites^40,41^, and the analysis did indeed result in the detection of four positive selection sites in the head domain of sH1N1 while a single site, aa468, was detected in the stalk domain (H1 numbering based on A/South Carolina/1/1918 starting with methionine) (Fig. 3A). Pandemic H1N1 had zero positive selection sites in the head and stalk domains. These negative results are likely due to the shorter sampling time frame^36^. The H3N2 head domain showed eleven positive selection sites while having zero positive selection sites in the stalk domain (Fig. 3B). The B/Victoria/2/87-like lineage had two positive selection sites in the head domain and no stalk positive selection sites. Finally, the B/Yamagata/16/88-like lineage showed one positive selection site in the head domain and no positive selection sites in the stalk domain (Fig. 3C). These results are summarized in Table 3. A few of the positive selection sites for H3N2 (138, 161 and 175) and B/Victoria (161) are predicted to be involved in N-glycosylation, which aids in the evasion of neutralizing antibodies, however aa468 was not, suggesting it is not involved in this type of immune evasion. The relative scarcity of positively selected sites compared to other selection analyses might be the consequence of a very stringent screening model. Additionally, the FEL method has been known to show fewer true positives with larger data sets, a tradeoff of having less false positives^39^.

**Table 3:** Summary of selection analyses. Values are an average of 3 subsamples.

Virus	Amino Acid (H1)	dN	p-value	Location
sH1N1	203	2.8	0.058	Head Domain (Sb)
	204	7.6	0.001	Head Domain (Sb)
	239	5.9	0.0001	Head Domain (Ca2)
	278	1.5	0.034	Head Domain
	468	1.9	0.032	Stalk Domain
pH1N1	no values were found in 3/3 subsamples			
H3N2	137	2.0	0.045	Head Domain (Site D)
	153	3.0	0.002	Head Domain (Site A)
	154	2.3	0.012	Head Domain (Site A)
	158	2.4	0.024	Head Domain (Site A)
	161	5.4	0.001	Head Domain (Site A)
	175	2.0	0.033	Head Domain (Site B)
	204	1.0	0.038	Head Domain (Site B)
	209	2.7	0.023	Head Domain (Site B)
	213	1.7	0.030	Head Domain (Site B)
	242	3.4	0.053	Head Domain (Site B)
	278	1.5	0.010	Head Domain (Site C)
B/Vic	161	3.0	0.007	Head Domain
	277	2.8	0.009	Head Domain
B/Yam	214	3.4	0.015	Head Domain

### Testing the effect of positive selection site aa468 on the neutralizing potency of human cross-reactive, anti-stalk monoclonal antibodies

**Figure 4:**
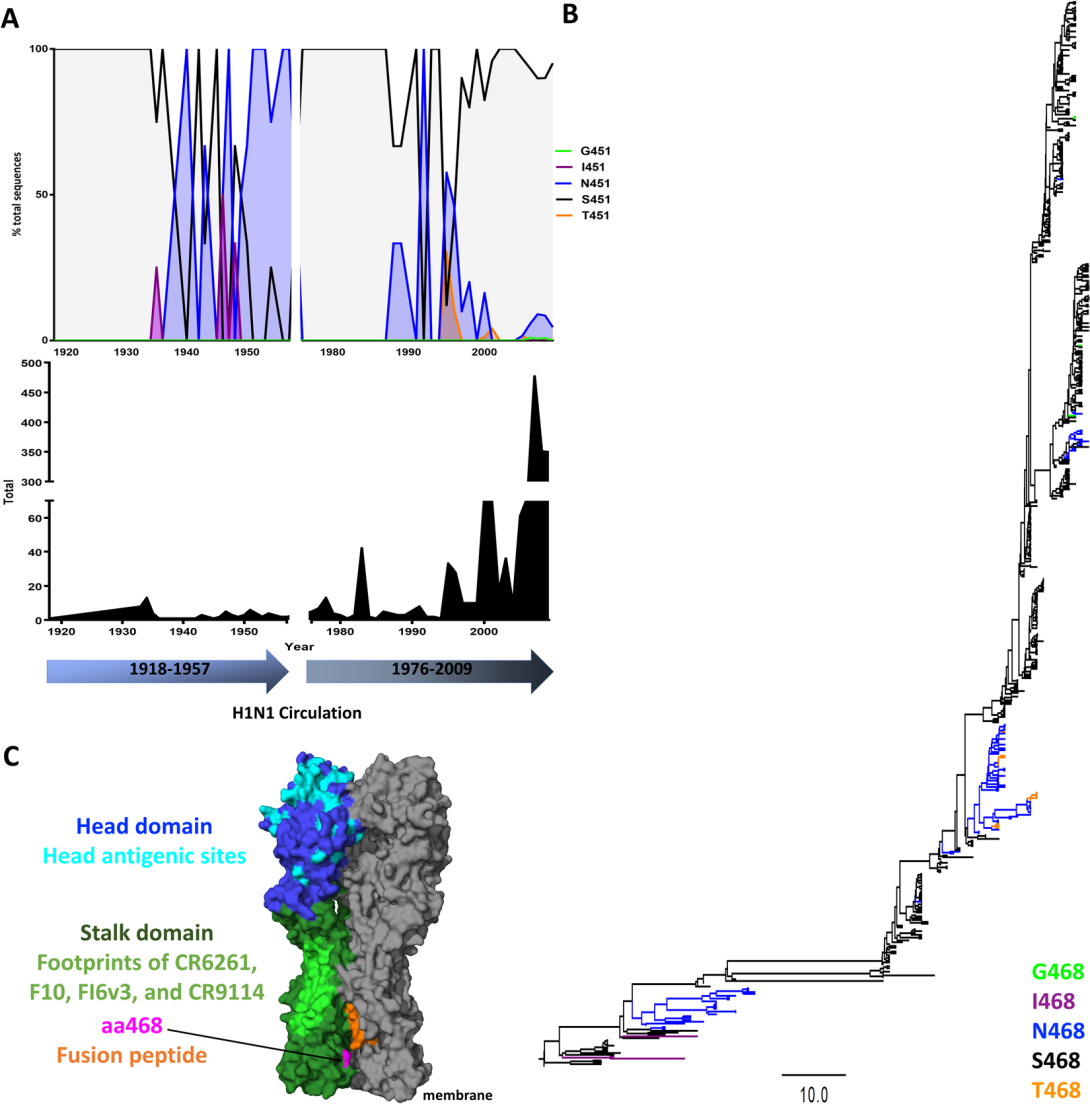
Location and phenotype of stalk positive selection site aa468 in sH1N1 influenza virus HAs. (**A**) Amino acid phenotype of aa468 during sH1N1 circulation. The bottom of the figure shows the timeline of circulation (1918–1957, 1976–2009). The total number of sequences used for the amino acid percentages is shown in the center while the percent of these sequences containing a specific amino acid at site 468 is shown at the top. The most prevalent amino acids in this position are serine (S468, in black) and asparagine (N468, in blue). These lines show that aa468 shifted between the two amino acids many times during circulation, including years of fixation of a particular amino acid or years of co-circulation. **(B)** Phylogenetic tree of stalk sequences of sH1N1 showing the amino acid 468 phenotype. S468 is in black and N468 is in blue. This tree illustrates the predominance of S468 in aa468 and the periodic fixation of N468 (1946, 1991) or the co-circulation of S468 and N468 in 2007. The scale bar shows the percent change at the nucleotide level. This tree is rooted to A/South Carolina/1/1918 and was generated using BEAST. **(C)** Three-dimensional representation of the location of aa468 on the sH1N1 HA (PDB ID 1RU7^74^). The head domain and antigenic sites are in blue and cyan, stalk domain and mAb footprints are in light and dark green, and the fusion peptide is in orange. Amino acid 468 is shown in magenta and indicated by an arrow. It is not located within the region of stalk cross-reactive mAb footprints but near the fusion machinery, hinting that its role may be in aiding in fusion of the HA.

**Figure 5:**
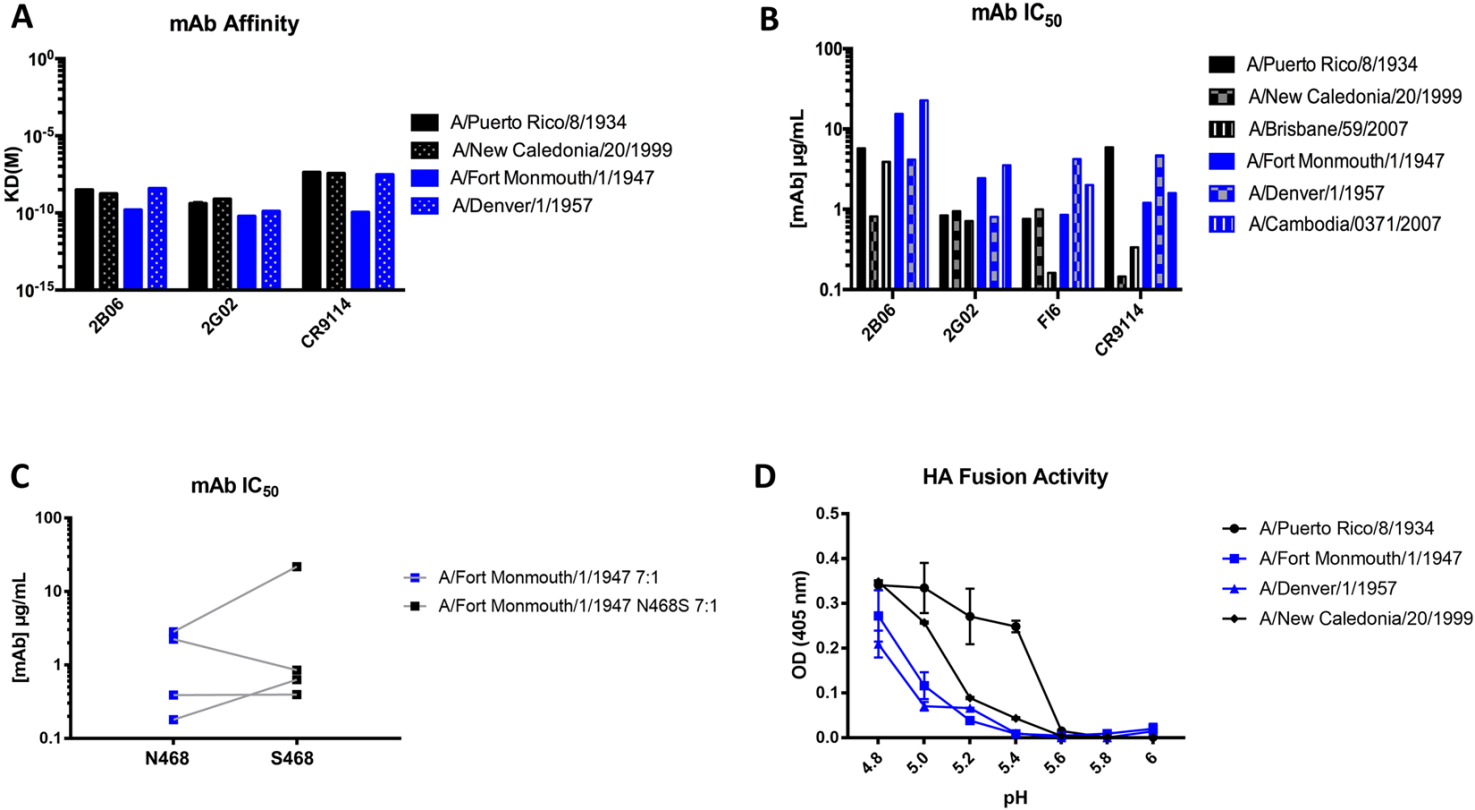
Characterizing viruses containing polymorphisms at aa468 with cross-reactive anti-stalk mAbs. (**A**) Affinity of mAbs to various sH1N1 viruses containing either an S468 (in black) or N451 (in blue). (**B**) IC_50_s of mAbs to various sH1N1 viruses containing S468 (in black) or N468 (in blue). (**C**) IC_50_s of reverse genetics virus containing seven A/Puerto Rico/8/1934 genomic segments and the wild type HA of A/Fort Monmouth/1/1947 (N) or a mutated version (S). The blue symbols represent the wild type N468 virus and the black represent N468S. Each square represents a single antibody (mAbs 2B06, 2G02, FI6, CR9114). The lines connect the data for the same antibody against the two viruses. (**D**) The HA fusion activity of purified viruses measured by detecting hemolysis via optical density (OD). Viruses containing a S468 are in black while viruses containing N468 are in blue.

The selection analysis showed that aa468 from sH1N1 was the only site in the stalk domain that seemed to be under positive selection, although the dN for this site was lower than for most positive selection sites in Table 3. We were curious if this amino acid was selected because of antigenic challenges, or for other purposes in HA structure and function. To assess aa468’s role, we investigated its location in the HA protein and its phenotype during sH1N1 circulation. As shown in Fig. 4C, aa468 is not located within the binding footprint of anti-stalk cross-reactive mAbs. In fact, the site is much closer to the fusion peptide of the HA. Over the approximately 90 years of circulation, aa468 shifted between 5 different amino acids, with a serine 468 (S468) and an asparagine 468 (N468) being the predominant phenotypes (Fig. 4A,B). S468 and N468 shift between one phenotype dominating the population over another (as seen in 1918–1935, 1950–1956, and 1976–1985) and co-circulation (as seen in 1935–1950 and 1985–2009). Although the N468 mutation appears several times over the course of sH1N1 evolution, it seems to die out each time. It is interesting that the serine, which we consider the wild type phenotype, has a random mutation potential to undergo six nonsynonymous mutations to become glycine (G), isoleucine (I), asparagine (N), serine (S), arginine (R), or threonine (T), but it seems to mostly undergo the single nucleotide change to create the N468 mutation. Because this occurs higher than at random chance and more than once in sH1N1 history, we wanted to investigate if this S468N mutation was occurring because of potential antigenic pressures to the HA stalk domain. We started this investigation by developing a panel of H1N1 viruses that circulated before 2009 and contained either an S468 or N468 (Table 4). We then selected four human mAbs based on their characterization as stalk specific and broadly reactive, as well as being able to bind and neutralize H1N1 viruses (Table 5). Binding experiments show that there is no significant difference between the affinity of each mAb to viruses containing S468 or N468 (Fig. 5A). To assess neutralizing potential of these mAbs to each virus, we conducted plaque reduction neutralization assays and measured the 50% inhibitory concentrations (IC_50_s) of each mAb/virus combination. The data show that the S468 and N468 containing viruses are neutralized similarly by the mAbs (Fig. 5B). While S468 viruses appear to have different binding affinities and to be slightly better neutralized by these mAbs than N468 viruses, this trend is not statistically significant indicating that the positive selection site in the stalk domain is not likely to be directly related to evading an antibody response. To further confirm these results, we used a reverse genetics system to generate recombinant A/Fort Monmouth/1/1947 viruses containing seven genomic segments from A/Puerto Rico/8/1934 in combination with the A/Fort Monmouth/1/1947 wild type HA (7:1 virus) or with A/Fort Monmouth/1/1947 N468S HA (N468S 7:1 virus). These viruses were also used in plaque reduction assays and while there were differences in virus neutralization, no general trend between the neutralizing efficiencies of the mAbs for wild type HA 7:1 virus versus the N468S virus was observed (Fig. 5C). Finally, to investigate if aa468 might play a role in HA fusion activity, we conducted HA fusion assays using purified viruses. These assays showed that the two tested S468 viruses triggered fusion at a higher pH than N468 viruses (Fig. 5D). While this data is only based on a comparison of 2 viruses per S/N phenotype it suggests that the choice of amino acid at position 468 might play a role in fine-tuning the fusion machinery.

**Table 4:** Virus panel for evaluating the effect of aa468 on neutralizing potencies of anti-stalk mAbs.

Virus Panel	aa468
A/Puerto Rico/8/1934	Serine (S)
A/Fort Monmouth/1/1947	Asparagine (N)
A/Denver/1/1957	Asparagine (N)
A/New Caledonia/20/1999	Serine (S)
A/Cambodia/0371/2007	Asparagine (N)
A/Brisbane/59/2007	Serine (S)

**Table 5:** Antibody panels for evaluating the effect of aa468 on neutralizing efficiencies of mAbs.

Antibody Panel	Source	Coverage
045-051310-2B06^53^	Human	Group 1 and Group 2
SFV005-2G02^77^	Human	Group 1 and Group 2
FI6v3^13^	Human	Group 1 and Group 2
CR9114^12^	Human	Group 1, Group 2, and influenza B virus HA

## Discussion

The evolution and selective forces that act on the head domain of the HA are relatively well studied^26,42–45^. However, less is known about the evolution of the stalk domain, which has become a major target for universal/broadly protective influenza virus vaccines. Our data clearly show that the stalk domains of H1, H3 and influenza B HAs are evolving at a significantly slower rate than the head domains of these HAs, verified by statistical supports from Bayes Factors and likelihood ratio tests. Quantitatively, this difference is even bigger when the head antigenic sites, which are the major target of the antibody response, are compared to the footprint of broadly neutralizing anti-stalk antibodies, which would likely be generated by universal/broadly protective vaccines. While the pattern of higher rates in the antigenic sites was consistent across all analyses, the empirical estimate should be treated with caution as this is based on a comparative analysis of a small section of the HA protein where mutations were concentrated. Regardless, the pattern of mutational rates in the head compared to the stalk is supported by our analysis of relative positive selection on the regions. Our findings are in agreement with other studies that found more diversifying selection in the head domain than in the stalk domain or found that the evolutionary rate of sites on the HA is positively correlated with their distance to the viral membrane (the further away, the faster they evolve)^43,46^. Other work has described how mutations in the stalk domain, specifically those involved in ‘universal antibody recognition sites’, confer low relative fitness compared to wild type amino acids, supporting our low evolutionary rates and relatively little positive selection in these regions^47^. In general, there are several explanations for our observation. There might not be enough immunological pressure on the stalk domain to drive evolution because antibody levels in humans are low^48,49^. In addition, stalk-reactive antibodies might be less potent than head reactive antibodies, which further reduces the effect of the already low antibody titers. This argument could be supported by the fact that while sH1N1 stalk evolution, including rates of stalk mAb footprints, was slower than H3N2 stalk evolution, the level of anti-H1 stalk-reactive antibodies is higher in humans than the level of anti-H3 stalk reactive antibodies^48,50^. Another explanation is that variants which have mutations in the stalk domain due to antibody pressure have low fitness, do not spread efficiently and are therefore not detected. This could introduce a “survivor bias” into the analysis, which is the idea that we observe the lack of selection or polymorphisms in a site because selection in this site leads to a less fit virus^51^. This idea is to some extent supported by data showing that many laboratory generated stalk escape mutants are not fit *in vivo*^52,53^. Additionally, there is evidence to support that the escape from broadly cross-reactive antibodies, for example FI6v3 or CR9114, is especially difficult. This may be because a single point mutation is often not enough to completely abolish binding^54,55^. As an example, passaging A/California/04/2009 H1N1 virus with up to 10 μg of mAb CR9114 caused the introduction of point mutations in the HA stalk domain, but it did not completely abolish mAb binding^55^. Furthermore, we found that influenza B virus HA evolution is slower than influenza A virus HA evolution for both the head and the stalk domains. Interestingly, the evolutionary rate of the stalk for influenza A virus HAs is inversely correlated with how long the respective virus clade is already circulating in the human population. Seasonal H1N1 (entered the population in 1918) displayed the lowest rate followed by H3N2 (1968) followed by pH1N1 (2009) with the head domains evolving at almost the same rate. This could indicate that features of the stalk - e.g. the fusion machinery or interactions that stabilize the HA trimer - have to initially adapt to the new human host. A potential example of this phenomenon is amino acid 374 (HA0 numbering) of pH1N1 which changed from E to K relatively early during the 2009 pandemic and conferred stability to the unstable trimer of the initial isolates^56^.

The relative conservation of the stalk domain might be due to its immunosubdominant nature (and hence the lack of antibody pressure), but it is also likely that this observation is caused by a lack of tolerance to changes due to the functional constraints of the fusion machinery. If the lower mutation rates are due to functional constraints, then there is a high likelihood that direct targeting of the stalk domain by universal influenza virus vaccines or mAb therapies would be sustainable options for influenza prevention and treatment. While amino acid positions in the head domains (close to or overlapping with antigenic sites) were found to be under positive selection for most analyzed HAs, only one site in the stalk was detected. This site (468) was only detected for sH1N1. Of note, this site was also identified to be under positive selection in pH1N1 HA by an earlier study^46^. Two major phenotypes, S and N, exist in position 468 for sH1N1 with N appearing occasionally and then disappearing again and S being the main phenotype that perpetuates new clade formation (Fig. 4B). Additionally, early pH1N1 isolates had an S in position 468 which changed to N over time^46^. The site is located close to the fusion peptide and slightly hidden within the trimer interface but is distant from the epitope of major stalk-neutralizing mAbs. Our data suggests that the polymorphism has no major impact on binding or neutralization by human stalk-reactive antibodies and might be related to changing HA fusion activity. Therefore, the selective pressure on this site might not be directly related to the human antibody response. While our stringent selection analyses uncovered fewer results than previous work, it was also more selective than those done previously^39,41,44–46,57^. We chose to look at sites that had a high number of polymorphisms at a given amino acid, not including those undergoing periodic evolution or abrupt fixation. Choosing this type of selection method allowed for the evaluation of overall flexibility in the hemagglutinin head and stalk domains, and was a suitable complement to the evolutionary analyses that would evaluate how well the domains adapt to antibody pressures^57^.

In any case, the analysis conducted here is based on virus sequences from the past. Any predictions about stalk evolution under enhanced pressure from anti-stalk immunity based on this data would therefore be speculation. However, the tolerance of the stalk domain to changes has been recently tested experimentally with two independent systems which either introduce five amino acid insertions or random mutations^44,45,58^. While the two experimental setups were radically different they both came to the conclusion that the stalk domain is highly intolerant of changes. This might be explained by the function of the stalk during virus replication, where this domain completely refolds to induce fusion of viral and host endosomal membranes. Taken together the current data suggests that the stalk domain is highly conserved, relatively intolerant to changes and evolving at a slower amino acid substitution rate than the head domain, making it a superior target for broadly protective/universal influenza virus vaccines.

## Materials and Methods

### Sequence Data Preparation

Data sets for influenza A viruses were created using human isolate sequences from the Influenza Research Database (https://www.fludb.org/) and were sorted to exclude all laboratory strains and duplicate sequences. The sequences were downloaded as a FASTA file containing: accession number, sequence ID, country of origin, and date of collection for each sequence. Data sets for influenza B viruses were created using human isolate sequences from the Global Initiative on Sharing All Influenza Data (http://platform.gisaid.org/) and were sorted based on belonging to either the B/Victoria/2/87-like or B/Yamagata/16/88-like lineages. Once downloaded, any sequences that had a passage number above 1 were removed, along with incomplete sequences. The data sets were then sorted by date and separated by year. All data sets were aligned using MUSCLE^59^. The alignments were then manually optimized to remove the non-coding regions before and after the HA protein sequence^60^. The final data sets contained 1511 sequences for sH1N1, 10015 sequences for pH1N1, 10331 sequences for H3N2, 1981 sequences for B/Victoria/87-like, and 5810 sequences for B/Yamagata/88-like HAs and were used for subsequent analyses.

### Summary of the effects of passaging on overall nonsynonymous substitution rates for H1N1

It has been shown that passaging of virus isolates before sequencing can introduce signals of positive selection compared to non-passaged isolates^22^. Importantly, our sequences from the Influenza Research Database (IRD) do not have attached information on passage history while sequences from the Global Initiative on Sharing All Influenza Data (GISAID) were obtained from isolates with up to only 1 passage. We generated data sets of “pooled” sequences, which could be either passaged or non-passaged isolates, and strictly non-passaged isolate sequences to use for a subsequent dN correlation analysis, similar to McWhite *et al*.^22^. Using sequences from our final data sets of H1N1, we generated 3 independent “pooled” data sets that contained 219 sequences from 2006–2017. We also obtained sH1N1 sequences from GISAID and generated 3 independent “unpassaged” data sets containing 219 sequences from 2006–2017. The “unpassaged” data sets contained sequences with a passaging history of “original”, “swab”, “initial”, “direct”, or “clinical specimen”. These data sets were aligned and uploaded to the webserver Data Monkey for FEL selection analysis. The.csv generated by Data Monkey was downloaded and each codon dN mean value (n = 3) was compared between the unpassaged and pooled datasets. We first looked at correlating the mean dN values for each codon between the two datasets and found a correlation coefficient of 0.7633 (Fig. S3A). We additionally looked at the overall mean dN values for each dataset and found that while the pooled samples have a slightly higher mean and larger spread of values than the unpassaged samples, the two means are statistically similar in a Student’s t-test (Fig. S3B).

### Rate variation among coding regions

Whole data sets were subsampled by year, using a random number generator, to contain a total number of sequences that was less than 1000 but also to ensure a minimum of 35 sequences per year (if available). Preliminary maximum likelihood phylogenetic trees were generated with RAxML^61^. Datasets were subsequently screened to remove identical sequences where the oldest unique sequence was maintained for subsequent analyses. The temporal signal was investigated using the ML trees produced above with TempEst v1.5^62^. Sequences that fell outside the residual spread of -0.005 and 0.005 or produced extremely long-branch lengths were removed for subsequent analyses. The final data sets contained 577 sequences for sH1N1, 431 sequences for pH1N1, 738 sequences for H3N2, 288 sequences for B/Victoria/87-like, and 252 sequences for B/Yamagata/88-like. These alignments can be found on GitHub (https://github.com/KrammerLab/Kirkpatricketal2018Supplementary). Bayesian phylogenetic trees were estimated using BEAST v.1.8.4^63^ with an uncorrelated lognormal relaxed molecular clock^64^ that allows for rate variation across lineages. Exact date of isolation was used to calibrate the clock. In cases where the exact date was only known to month, we assigned the date to represent the middle of the month and when only the year was known we used middle of the year to represent the date of isolation. A GMRF Bayesian Skyride coalescent tree prior was chosen^65^ to account for oscillations in the demographic history of the viral populations. To estimate domain specific rate variation we used a structurally informed evolutionary model where a nucleotide substitution patterns could be co-estimated for each conserved domain^23^. Partitioning for the head domain was done to include the entire head from C59 to C292 for H1N1 viruses, C68 to C293 for H3N2 viruses, and the respective A57 and A306 for influenza B viruses (Fig. 2A–C). The stalk domain includes two portions, from D18 to L58 (the N-terminus of HA1) and N293 to Q529 (the C-terminus of HA1 plus the ectodomain of HA2) for H1N1 viruses, Q17 to I67 and I294 to W530 for H3N2 viruses, and D16 to F56 and D307 to T548 for influenza B viruses. The head and stalk domains were determined based on a multiple sequence alignment to the A/South Carolina/1/1918 strain for H1N1, A/Aichi/2/1968 for H3N2, and B/Yamagata/16/1988 for influenza B viruses and their previously defined regions, with numbering beginning from methionine^24^ For the head and stalk partitions, we applied the SRD06 approximate codon model (which applies a Hasegawa-Kishono-Yano (HKY) model to codon position 1 + 2 and a HKY model to position 3, separately) and for the STC region we applied an HKY model. This approach allows the substitution rate to vary according to its protein structure rather than unrealistically assuming a single rate across the entire gene. The likelihood is jointly estimated for each domain, given a single tree^66^. The Bayesian simulation integrates the posterior likelihood across all possible trees to account for phylogenetic uncertainty. A uniform prior was applied to the relative rate parameter ranging from 0 to 1E100. The MCMC was set to 100 million generations sampled every 10,000 steps and repeated 4 times.

### Substitution rate estimation of important antigenic and epitope sites

A sampling of 500 trees selected from the output generated above was used as an empirical set in order to estimate evolutionary rates of the head antigenic sites or the approximate stalk mAb footprints of F10, CR6261, CR9114 and FI6v3 (for H1 viruses) or CR8043, CR8020 (residue R25 in HA2 numbering was omitted from the CR8020 and CR8043 epitopes), CR9114, MEDI8852 and FI6v3 for H3 viruses)^12–14,20,26,29^. The data was analyzed under the amino acid substitution model FLU^67^ with a strict clock model since only a portion of the gene was analyzed. The amino acids were mapped onto the nucleotide trees and the amino acid substitution rates for each antigenic site or mAb was estimated from repeated sampling of this empirical tree space. This analysis was repeated three times, each time with a new set of 500 randomly selected trees. The MCMC was set to 1 million with a log every 100. The three runs were combined and the AA substitution rate estimates summarized.

### Data Monkey Analyses

Selection analysis was conducted using the web server Data Monkey (http://www.datamonkey.org)^37,38^. The data sets were randomly sub-sampled by year, using a random number generator, so that the total number of sequences in each sub-sample was less than 500 but also ensured a minimum of 20 sequences per year (if available). This sub-sampling was done three times per data set and all three subsamples were used for selection analysis. Each sub-sample was uploaded to Data Monkey with a respective maximum likelihood tree. All three datasets, along with their maximum likelihood trees are available online at GitHub (https://github.com/KrammerLab/Kirkpatricketal2018Supplementary). First, each sub-sample was analyzed by the web server to determine the model of nucleotide substitution to use for each data set. Next, the files were uploaded again and analyzed using the fixed-effects-likelihood (FEL) method, with a significance level (defined as p-value/Bayes Factor/posterior probability) set to 0.1, using the user defined tree^39^. This analysis was run on each sub-sampled set of sequences for each virus dataset. Only positive selection sites detected in all three of the subsamples were included in the results.

### Determining Antibody Affinity

HAs of H1N1 strains A/Puerto Rico/8/1934, A/Fort Monmouth/1/1947, A/Denver/1/1957, and A/New Caledonia/20/1999 were produced using a baculovirus expression system^68^. Affinity (K_D_) was determined by biolayer interferometry using the FortéBio Octet QK^e^ system and anti-human IgG Fc capture (AHC, catalog number 18–5064) biosensors. The experimental setup was similar to previously published work^69^. Sensors were first incubated for 180 seconds (s) with 1X kinetics buffer (1X phosphate buffered saline (PBS, pH 7.4), 0.01% bovine serum albumin (BSA), 0.002% Tween 20) to establish a baseline measurement. The sensors were then incubated in 20 μg/mL of respective mAb, in 1X kinetics buffer, for 180 s to allow for association of mAb. To measure *k*_on_, the sensors were then incubated in 200 μL of eight three-fold dilutions (300 μg/mL–0.14 μg/mL) of rHA proteins, in 1X kinetics buffer, to allow for rHA-mAb association. Finally, to measure *k*_off_, the sensors were incubated in the baseline 1X kinetics buffer for 180 s to allow for rHA-mAb dissociation. Experiments were perfomed at 30 °C and the K_D_ ratio was determined using the Octet software, which compares the ratio of *k*_off_ to *k*_on_.

### Plaque Reduction Neutralization Assay

Virus stocks of A/Puerto Rico/8/1934, A/Fort Monmouth/1/1947, A/Denver/1/1957, A/New Caledonia/20/1999, A/Cambodia/0371/2007 and A/Brisbane/59/2007 were grown in 10-day old specific pathogen-free (SPF) fertilized chicken eggs. Plasmids encoding human mAbs were obtained from Dr. Patrick Wilson (University of Chicago)^70^. Antibody was generated by transfecting Expi293F cells and purifying cell culture supernatant using a protein G column^71^. Cells were transfected following the standard Expi293F protocol (ThermoFisher catalog number A14515) in 30 mL of ExpiFectamine 293 media for seven days. Plaque reduction assays were conducted as described previously^71,72^. Briefly, five five-fold dilutions of mAbs were generated with a starting concentration of 100 μg/mL (100 μg/mL–0.032 μg/mL). These mAb dilutions were then incubated with the respective virus for one hour at room temperature in 1X minimal essential medium (MEM; 10% 10X MEM, 1% 200mM L-glutamine, 1.6% of a 7.5% sodium bicarbonate stock solution (pH 7.5), 1% of a 1 M 4-(2-hydroxyethyl)-1-piperazineethanesulfonic acid (HEPES) stock solution, 1% of penicillin/streptomycin antibiotic cocktail (Pen/Strep, Gibco), 0.6% of a 35% BSA stock solution), shaking. The virus/antibody mixture was then incubated on a confluent monolayer of Madin Darby Canine Kidney (MDCK) cells (ATCC number PTA-6500; maintained in complete Dulbecco’s Modified Eagle Medium (DMEM with the addition of 10% fetal bovine serum (FBS), 1% Pen/Strep, 1% of a 1 M HEPES stock solution) for 40 minutes at 37 °C. Then the infection media was removed and replaced with an agarose overlay (containing 2X MEM, 6.25% diethylaminoethanol, 0.625% 1mg/mL L-1-tosylamide-2-phenylethyl chloromethyl ketone (TPCK)-treated trypsin and 2% agarose) that contained mAb at the same dilution factor as the infection media. Infected cells were incubated for 48 hours at 37 °C with 5% CO_2_ to allow for plaque formation. Then the plaques were visualized using immunostaining. First, cells were fixed with 3.7% paraformaldehyde at 4 °C overnight. The cells were then blocked with 3% non-fat milk for 1 hour. Primary antibody, KB2^24^ was diluted 1:1000 in 1% non-fat milk and incubated for one hour at room temperature, shaking. As a secondary horseradish peroxidase conjugated anti-mouse IgG antibody (Rockland, catalog number 610-4302) was used at a 1:3000 dilution and incubated for 30 minutes at 37 °C. Finally, cells were stained with TrueBlue Peroxidase Substrate (catalog number 50-78-02) for 20 minutes at room temperature (covered and shaking). Plaques were manually counted and compared to an irrelevant IgG control mAb plate to calculate percent inhibition of the virus by the antibody.

### Virus Rescue

Viruses with wild type HA (N) or mutated HA (S) from A/Fort Monmouth/1/1947 were rescued using a previously described reverse genetics system^73^. To amplify the A/Fort Monmouth/1/1947 wild type HA the primers TTTTGGGCCGCCGGGTTATTAGTAGAAACAAGGGTGTTTTTCCTCATATTTCTGAAATTCTAATCTCAGATGCATATTCTGCATTGCAAAGACCC and TCGACCTCCGAAGTTGGGGGGGAGCAAAAGCAGGGGAAAATAAAAACAACCAAAATGAAAGCAAAACTACTGATCCTGTTATGTGC were used. The amplified HA was then digested using SapI (New England Biolabs, R0569S) and ligated into the rescue vector pDZ. The N468S mutation in the A/Fort Monmouth/1/1947 HA was generated from the wild type HA product using the primers GCATTATTCCTTAATTGGCTTTTTACTTTCTCATACAG and CTGTATGAGAAAGTAAAAAGCCAATTAAGGAATAATGC in combination with the above primers. The HA-pDZ plasmids were combined with 7 pDZ plasmids containing the remaining wild-type A/Puerto Rico/8/1934 genomic segments (NA, NS, PB1, PB2, PA, M, and NP) and transfected into 293 T cells (ATCC^®^ CRL-3216^™^) at a concentration of 1 µg each and incubated overnight at 37 °C. Cell supernatant was then injected into 8-day old specific pathogen-free (SPF) fertilized chicken eggs and incubated for 48 hours at 37 °C. The rescue viruses were then plaque purified, sequenced, and used in plaque reduction neutralization assays.

### Fusion Assays

Fusion assays were performed with purified virus. Virus was purified using NTE buffer (100 mM NaCl, 10 mM Tris, 1 mM EDTA, 30% sucrose) from allantoic fluid from 10-day old specific pathogen-free (SPF) fertilized chicken eggs containing either A/Puerto Rico/8/1934, A/Fort Monmouth/1/1947, A/Denver/1/1957 or A/New Caledonia/20/1999 virus. The allantoic fluid was ultra-centrifuged through a 30% sucrose/NTE cushion at 25,000 RPM in a Beckman SW28 rotor for 2 hours at 4 °C and the pellet resuspended in 1xPBS (Gibco). The HA activity of each virus preparation was determined in an HA assay. Briefly, virus preps were serially (1:2) diluted in 0.15 M NaCl in a 96-well v-bottom plate and incubated with 0.5% chicken red blood cells (7201403, Lampire Biological Laboratories) for 40 minutes at 4 °C. All viruses were then diluted to 128 HAUs for the fusion assays. Fusion assays were performed by incubating 128 HAUs of each virus in 0.15 M NaCl in a 96-well round-bottom plate with chicken red blood cells and allowing the mixture to hemagglutinate for 40 minutes at 4 °C. Then, 4 times the diluted virus volume of 0.15 M sodium citrate at varying pHs (4.8, 5.0, 5.2, 5.4, 5.6, 5.8, and 6.0) was added and incubated at 37 °C for 90 minutes. Each plate was spun at 800 g for 5 minutes at 4 °C and then 100 µL from each well was transferred into a 96-well flat-bottom plate and the optical density (OD) was read at 405 nm to detect hemolysis. The OD minus the background per plate/pH condition was reported.

### Statistical Analysis

Statistical analysis was conducted using Prism 6 (GraphPad software). Antibody affinities and IC_50_ values were compared for statistically significant differences using an unpaired, parametric Student’s t-test. The conditional probabilities used in the Bayes Factor Test to determine significance of rate variation in the head and stalk regions were calculated with Microsoft Excel.

### Data Availability

The data that support the findings of this study have partially been uploaded to github (as indicated above) and are available from the corresponding author upon request.

## Electronic supplementary material

Supplementary material
