# Supplementary material for: The influenza virus hemagglutinin head evolves faster than the stalk domain
Source: Sci Rep. 2018 Jul 11;8:10432. doi: 10.1038/s41598-018-28706-1 (PMC6041311; doi:10.1038/s41598-018-28706-1)
Supplement: Supplementary file 1 — Supplementary material [file 41598_2018_28706_MOESM1_ESM.pdf]

The influenza virus hemagglutinin head evolves faster than the stalk domain

Ericka Kirkpatrick[1,2], Xueting Qiu[3], Patrick C. Wilson[4], Justin Bahl[3,5]*, Florian Krammer[1]*

[1]*Department of Microbiology, Icahn School of Medicine at Mount Sinai, New York, NY*

[2]*Graduate School of Biomedical Sciences, Icahn School of Medicine at Mount Sinai, New York, NY.*

[3]*University of Texas School of Public Health, Houston, TX*

[4]*Department of Medicine, Section of Rheumatology, Gwen Knapp Center for Lupus and Immunology Research, University of Chicago, Chicago, IL*

[5]*Program in Emerging Infectious Diseases, Duke-National University of Singapore Graduate Medical School, Singapore*

Figure S1: Nucleotide based evolutionary rates of the influenza virus hemagglutinin head and stalk domains. (A) Linear schematic of the HA molecule and partitions used for analysis. The stalk domain amino acid demarcations are shown on the figure for H1 and H3 on the left (black) and influenza B on the right (blue). **(B-C)** Evolutionary rates of influenza A virus or influenza B virus whole hemagglutinin (peach), stalk/transmembrane/cytoplasmic domains (STC) (grey), head (blue) and, stalk (green) domains. The mean (indicated by black dots) and 95% credible interval of BEAST runs (using a single dataset) are shown in nucleotide substitutions/site/year (n/s/t).

Figure S2: Visualizing numbering for partitions. (A-C) Multiple sequence alignments of H1N1 (A), H3N2 (B), or influenza B (C) virus HAs. The signal peptide, transmembrane, and cytoplasmic domain (STC) are shown in grey, the stalk domain is shown in green, and the head domain is shown in blue. For H1N1 and H3N2 viruses, the amino acids specifically used in our evolutionary rate analyses of the head antigenic sites and stalk mAb epitopes are highlighted.

Figure S3: Summary of the effects of passaging on overall nonsynonymous substitution rates for H1N1. (A) Correlation of the mean dN for each codon between unpassaged and pooled data sets. Each data set codon dN is an average of 3 independent FEL selection analyses. The correlation coefficient is reported in the figure (0.7633). (B) Comparison of overall difference in mean dN between unpassaged and pooled (n=3 for each group). There is no significant (ns) difference between means.

Table S1: Summary of likelihood ratio tests. These tests were conducted on the non-partitioned, whole HA sequences versus the partitioned (STC, head, stalk) HA sequences. The significance indicates the probability of the partitioned model performing better than the non-partitioned model.

			**LRT statistics**	**degrees of freedom**	**critical value for chi-square with significance level =0.001**	**P-value for supporting Partitional model**
sH1N1	**nucleotide model**					
	Partitioned Log(Likelihood)	Non-partitioned Log(Likelihood)				
	-19409.315	-20214.554	1610.478	2	13.816	P-Value < 0.00001
	**codon model**					
	Partitioned Log(Likelihood)	Non-partitioned Log(Likelihood)				
	-18845.1	-19656.2	1622.2	2	13.816	P-Value < 0.001
			**LRT statistics**	**degrees of freedom**	**critical value for chi-square with significance level**	**P-value for supporting Partitional model**

					**=0.001**	
pH1N1	**nucleotide model**					
	Partitioned Log(Likelihood)	Non-partitioned Log(Likelihood)				
	-13923.459	-14551.487	1256.056	2	13.816	P-Value < 0.00001
	**codon model**					
	Partitioned Log(Likelihood)	Non-partitioned Log(Likelihood)				
	-13428.9	-14060.8	1263.8	2	13.816	P-Value < 0.001
	Partitioned Log(Likelihood)	Non-partitioned Log(Likelihood)	1383.106	2	13.816	P-Value < 0.00001
	-19064.542	-19756.095				
	**codon model**					
	Partitioned Log(Likelihood)	Non-partitioned Log(Likelihood)				
	-18497.1	-19183.5	1372.8	2	13.816	P-Value < 0.001
B/Vic	**nucleotide model**					
	Partitioned Log(Likelihood)	Non-partitioned Log(Likelihood)				
	-10027.183	-10410.123	765.88	2	13.816	P-Value < 0.00001
	**codon model**					
	Partitioned Log(Likelihood)	Non-partitioned Log(Likelihood)				
	-9527.88	-9912.66	769.56	2	13.816	P-Value < 0.001
B/Yam	**nucleotide model**					
	Partitioned Log(Likelihood)	Non-partitioned Log(Likelihood)				
	-9291.626	-9633.68	684.108	2	13.816	P-Value <

						0.00001
	**codon model**					
	Partitioned Log(Likelihood)	Non-partitioned Log(Likelihood)				
	-8793.41	-9135.43	684.04	2	13.816	P-Value < 0.001

Table S2: Summary of evolutionary rates of HA head and stalk domains of sH1N1, pH1N1, H3N2, B/Yamagata/16/88-like and B/Victoria/2/87-like viruses based on a functional partitioning model. Bayes factor is defined as Pr(Head>Stalk)/Pr(Stalk>Head) divided by the prior (~1).

**Virus**	**Mean Whole HA Evolutionary Rate (n/s/y)**	**Mean STC Evolutionary Rate (n/s/y)**	**Mean Head Evolutionary Rate (n/s/y)**	**Mean Stalk Evolutionary Rate (n/s/y)**	**Bayes Factor**
**sH1N1**	2.46×10^{-3}	2.18×10^{-3}	3.56×10^{-3}	2.42×10^{-3}	infinite
**pH1N1**	4.35×10^{-3}	4.00×10^{-3}	5.64×10^{-3}	5.18×10^{-3}	7.1
**H3N2**	4.16×10^{-3}	2.55×10^{-3}	4.76×10^{-3}	3.73×10^{-3}	infinite
**B/Vic**	2.80×10^{-3}	1.89×10^{-3}	2.98×10^{-3}	2.70×10^{-3}	7.7
**B/Yam**	2.73×10^{-3}	1.58×10^{-3}	3.20×10^{-3}	2.37×10^{-3}	infinite

Fig. S1

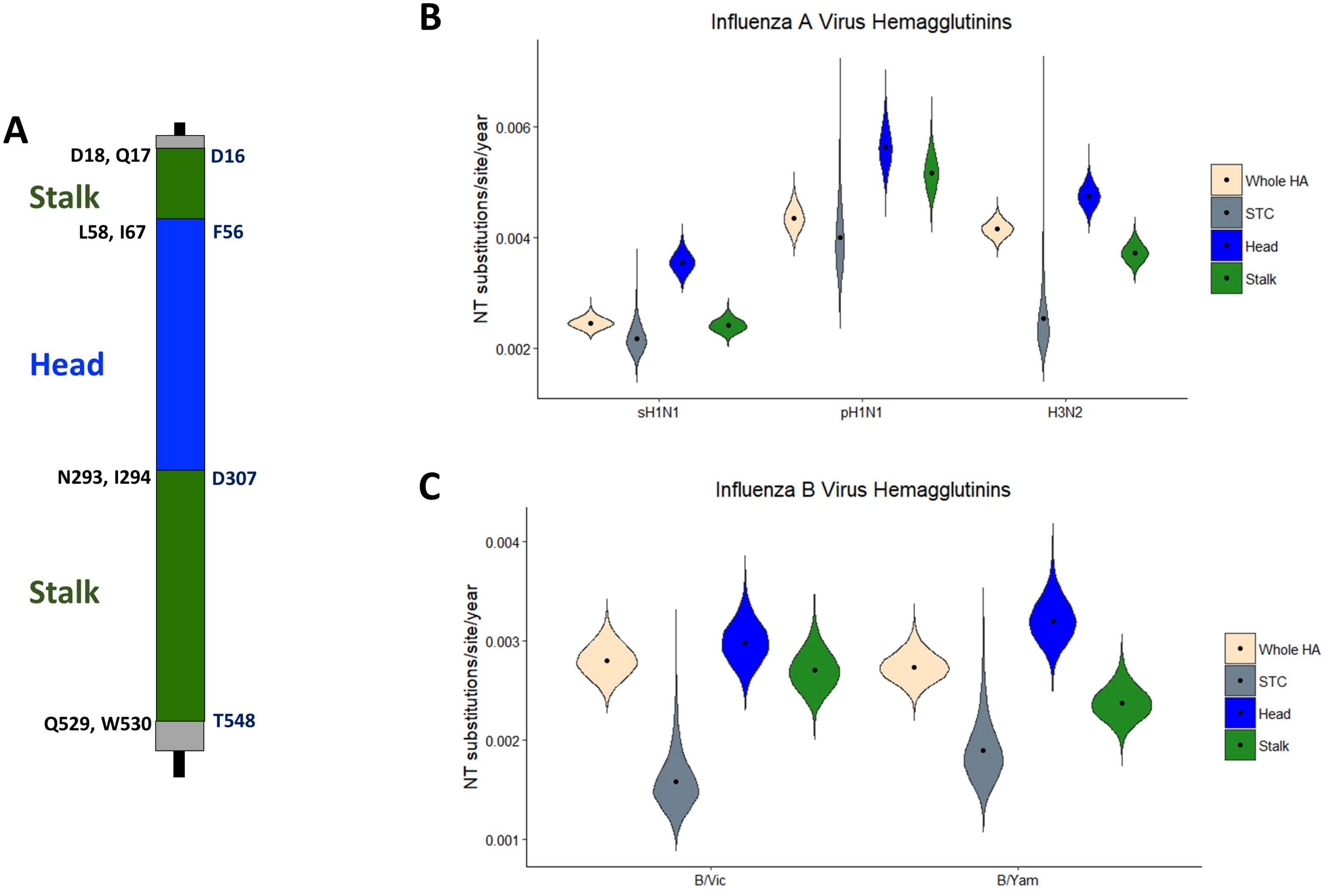
A
D18, Q17
D16
Stalk
L58, I67
F56
Head
N293, I294
D307
Stalk
Q529, W530
T548
B
Influenza A Virus Hemagglutinins
NT substitutions/site/year
0.006
0.004
0.002
sH1N1
pH1N1
H3N2
Whole HA
STC
Head
Stalk
C
Influenza B Virus Hemagglutinins
NT substitutions/site/year
0.004
0.003
0.002
0.001
B/Vic
B/Yam
Whole HA
STC
Head
Stalk

Fig. S2

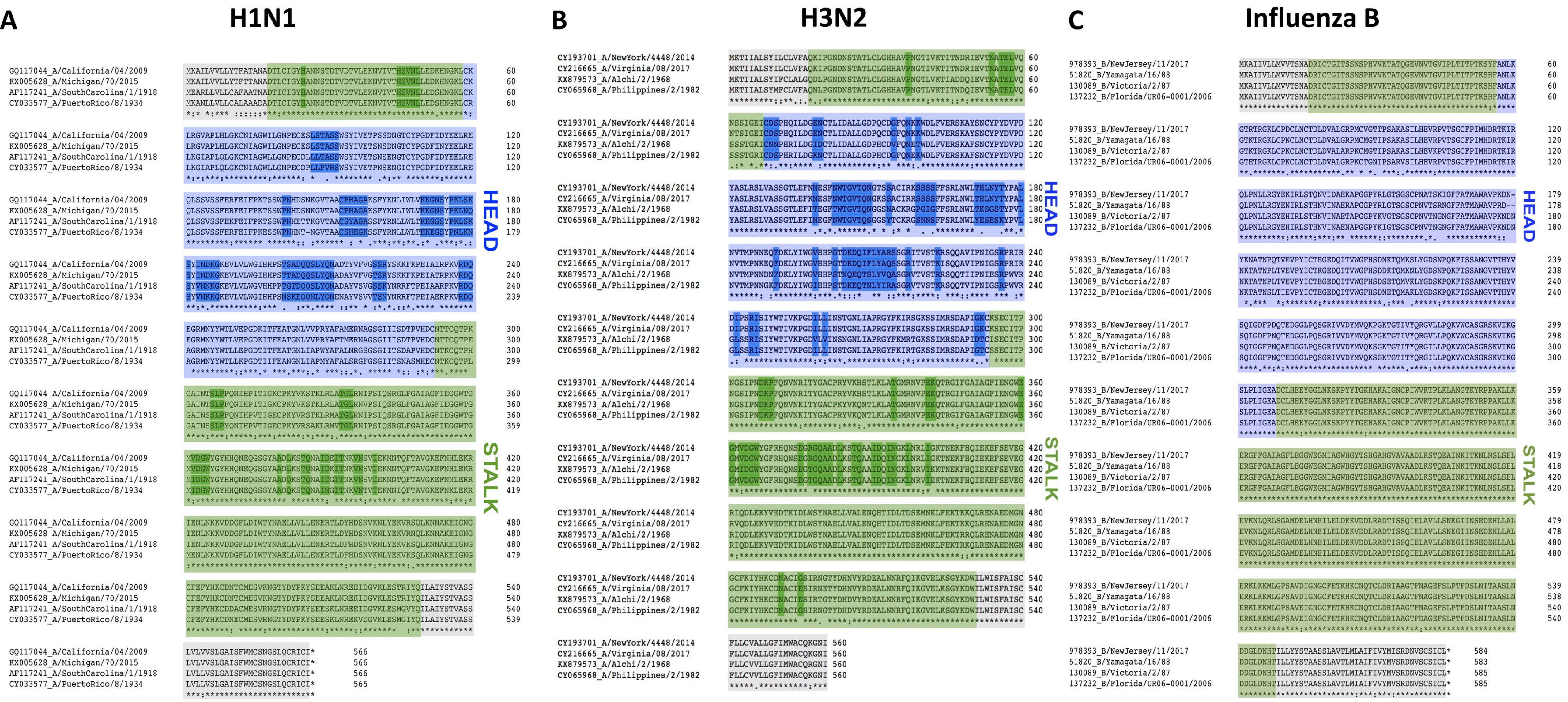
A
H1N1
GQ117044_A/California/04/2009
KX005628_A/Michigan/70/2015
AF117241_A/SouthCarolina/1/1918
CY033577_A/PuertoRico/8/1934
HEAD
STALK
B
H3N2
CY193701_A/NewYork/4448/2014
CY216665_A/Virginia/08/2017
KX879573_A/Alchi/2/1968
CY065968_A/Philippines/2/1982
HEAD
STALK
C
Influenza B
978393_B/NewJersey/11/2017
51820_B/Yamagata/16/88
130089_B/Victoria/2/87
137232_B/Florida/UR06-0001/2006
HEAD
STALK

Fig. S3

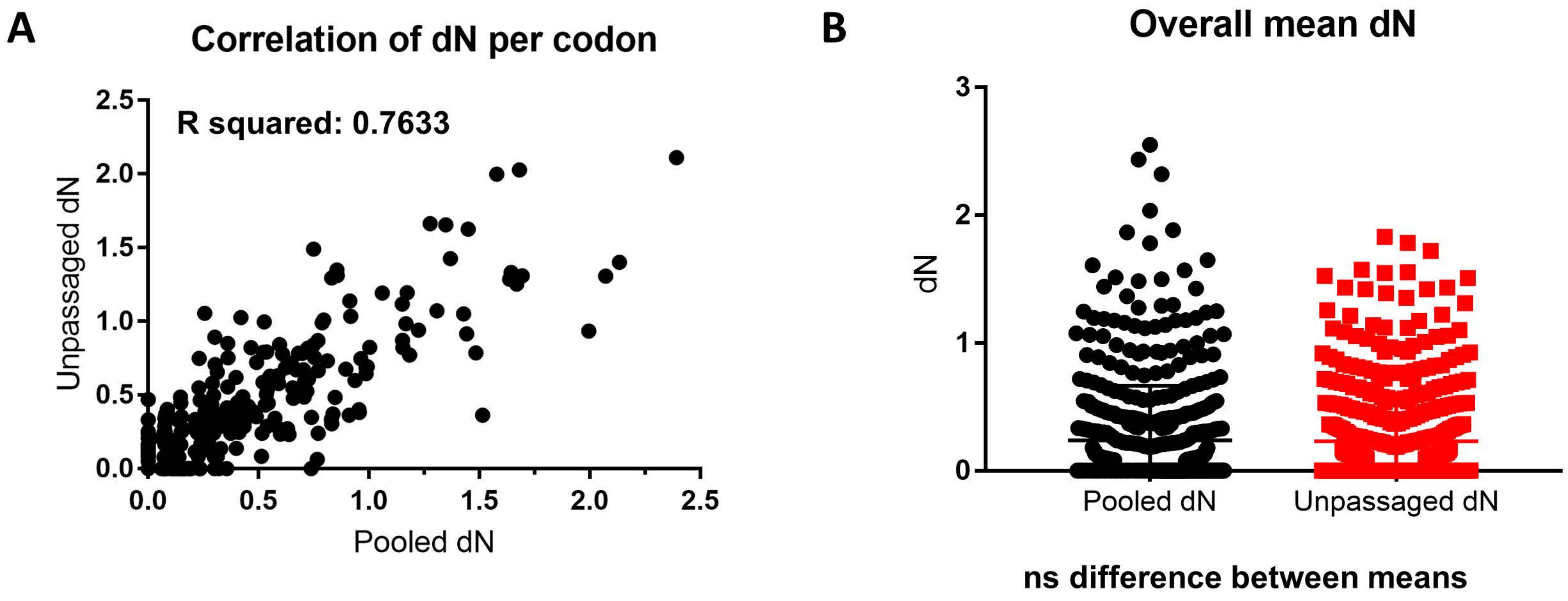
A
Correlation of dN per codon
R squared: 0.7633
Unpassaged dN
2.5
2.0
1.5
1.0
0.5
0.0
0.0
0.5
1.0
1.5
2.0
2.5
Pooled dN
B
Overall mean dN
dN
3
2
1
0
Pooled dN
Unpassaged dN
ns difference between means
